# Supine sleep patterns as a part of phenotyping patients with sleep apnea—a pilot study

**DOI:** 10.1007/s11325-022-02567-5

**Published:** 2022-01-12

**Authors:** Wojciech Kukwa, Jonasz Łaba, Tomasz Lis, Krystyna Sobczyk, Ron B. Mitchell, Marcel Młyńczak

**Affiliations:** 1 Department of Otorhinolaryngology, Faculty of Dental Medicine, Medical University of Warsaw, 19/25 Stepinska Street, 00-739 Warsaw, Poland; 2 Faculty of Mechatronics, Institute of Metrology and Biomedical Engineering, Warsaw University of Technology, Warsaw, Poland; 3 Department of Pediatric ENT, Medical University of Warsaw, Warsaw, Poland; 4 Department of Otolaryngology, UT Southwestern Medical Center, Dallas, TX USA

**Keywords:** Sleep position, Sleep apnea, Polysomnography, Home sleep apnea testing

## Abstract

**Purpose:**

Polysomnography (PSG) is considered the best objective study to diagnose and quantify sleep disorders. However, PSG involves multiple electrodes and is usually performed in a sleep laboratory that in itself may change the physiology of sleep. One of the parameters that can change during PSG is the sleep position, leading to more supine sleep. The aim of this study was to quantify the amount of supine sleep during PSG and compare it to consecutive nights of a home sleep apnea test (HSAT) in the same patients.

**Methods:**

This prospective study evaluated 22 consecutive patients undergoing PSG followed by HSAT. Sleep position was analyzed during PSG and subsequently on 2 to 6 nights (mean 3.7 nights) at home, and the amount of supine sleep was recorded during each night.

**Results:**

Of 22 patients, there were 12 men (55%). The median age was 60.0 years for women and 45.5 years for men. Median proportion of supine sleep during PSG and HSAT was 61% and 26% (*p* < 0.001), respectively. Four “phenotypes” were identified according to their sleep position during PSG and HSAT, with 5 patients sleeping mainly supine during all nights, 7 patients sleeping mainly non-supine during all nights, 3 patients sleeping in different positions during each night, and 7 patients sleeping supine during PSG but non-supine at home, during HSAT.

**Conclusions:**

There is a higher proportion of supine sleep during PSG compared to home sleep. We identified a subgroup of patients who slept mainly supine during PSG and mainly non-supine during HSAT. PSG may overestimate OSA severity in a specific phenotype of patients.

## Introduction

Polysomnography (PSG) is the gold standard for the diagnosis of obstructive sleep apnea (OSA) in both children and adults [1]. Apart from breathing parameters, PSG monitors and records many body functions during sleep, including brain activity, eye movements, muscle activity, and heart rhythm. PSG usually involves an in-lab overnight study which lasts up to 8 h. Sleeping in an unfamiliar bed and environment during PSG while connected to multiple electrodes is unappealing to many patients. It may lead to disorders of sleep and distort the results of PSG. Furthermore a “first night effect” (FNE) may increase arousal and inhibit a sleep-initiation process [2, 3]. Some patients also report feeling constrained during PSG due to the presence of numerous leads and monitors resulting in more supine sleep than at home [4]. This phenomenon was first described by Metersky et al. in 1996 who showed, on 12 patients, that the amount of supine sleep was 56% greater during a PSG night than during a non-PSG night at the same sleep lab environment [5]. This phenomenon may lead to overdiagnosis of OSA severity as most people have more apneic episodes while sleeping supine. The phenomenon is further exacerbated in patients with positional OSA (pOSA). Multiple studies show that more than 50% of tested patients have pOSA meaning their apnea–hypopnea index (AHI) is at least twice as high when supine than non-supine [6, 7]. Several studies have emphasized the importance of body position in the pathogenesis of sleep apnea. Changes in body position during sleep may affect the upper airways’ anatomy and its collapsibility with supine sleep having the most negative impact [8]. The importance of sleep position on OSA severity was recently highlighted by Ravesloot et al. who presented specific recommendations on how to report disease severity linked to various sleeping positions [9].

PSG is not the only way to diagnose sleep apnea. There are numerous abbreviated sleep studies, generally called home sleep apnea tests (HSAT). Portable monitors used in HSAT are limited channel devices classified into type III and type IV by the American Academy of Sleep Medicine. The effects of PSG and different types of HSAT on sleep physiology are scarce, despite the high likelihood that the device used may affect the sleep study results. The sleep position and the amount of supine sleep are the parameters which may be affected the most [4]. Therefore, we aimed to analyze the effect of in-lab PSG on the amount of supine sleep. The sleep position was measured during a PSG night and compared to home sleep while using a simple audio-motion HSAT (Clebre, Warsaw, Poland). Clebre is an 18-g sensor which is attached to the neck skin in the suprasternal notch with a double-sided medical patch (see “Methods”). Several previous studies have been published using Clebre that confirmed its ease of use and accuracy [10, 11]. The relative simplicity of Clebre allowed for a minimal interruption in the sleep physiology and for multiple sleep studies at home to be compared to a single in-lab PSG.

The primary objective of the study was to determine the effect of in-lab PSG on the amount of supine sleep. The secondary objective was to identify sleep position patterns during PSG and home sleep.

## Materials and methods

### Participants

All participants signed an informed consent. The study was approved by the Ethics Committee of Medical University of Warsaw (KB/14/2018). The study included consecutive adult patients who underwent PSG from March to September 2020. The inclusion criteria were 18 years of age or older, PSG requested for suspected OSA, at least 6 h of PSG recordings, and at least two recordings from HSAT. The exclusion criteria were a previous history of OSA treatment such as positive airway pressure (PAP) therapy and class III and IV heart failure according to the classification of the New York Heart Association (NYHA) [12].

### Protocol and devices

Demographic information including age, sex, height, and weight was collected. Body mass index (BMI) was calculated for each patient. All participants completed a Polish translation of the Epworth Sleepiness Scale (ESS) [13] and a PSG in the sleep laboratory of the Otorhinolaryngology Department at Czerniakowski Hospital, Warsaw, Poland. A standard PSG montage (Nox A1 PSG System, Nox Medical, Iceland) was used with 6-channel encephalogram (EEG), 3-channel submental electromyogram (EMG), left and right electrooculogram (EOG), electrocardiogram (ECG), airflow recording through the nose and mouth by a nasal air pressure transducer and oronasal thermistor, and thoracic and abdominal excursion measurements by inductance plethysmography and arterial oxygen saturation using Nonin 3150 WristOx2™ wireless oximeter (Nonin Medical, Plymouth, MN, USA). In addition, PSG headbox incorporated microphone, which was used to record snoring and a 3-axis, ± 2-g accelerometer with 10-Hz sampling frequency, to assess body position. The parameters were measured and recorded continuously and supervised by a polysomnographic technician. Sleep and respiratory events were scored by a sleep physician using criteria recommended by the American Academy of Sleep Medicine (AASM) [1]. The sleep position was obtained from the PSG sleep report.

**Fig. 1:**
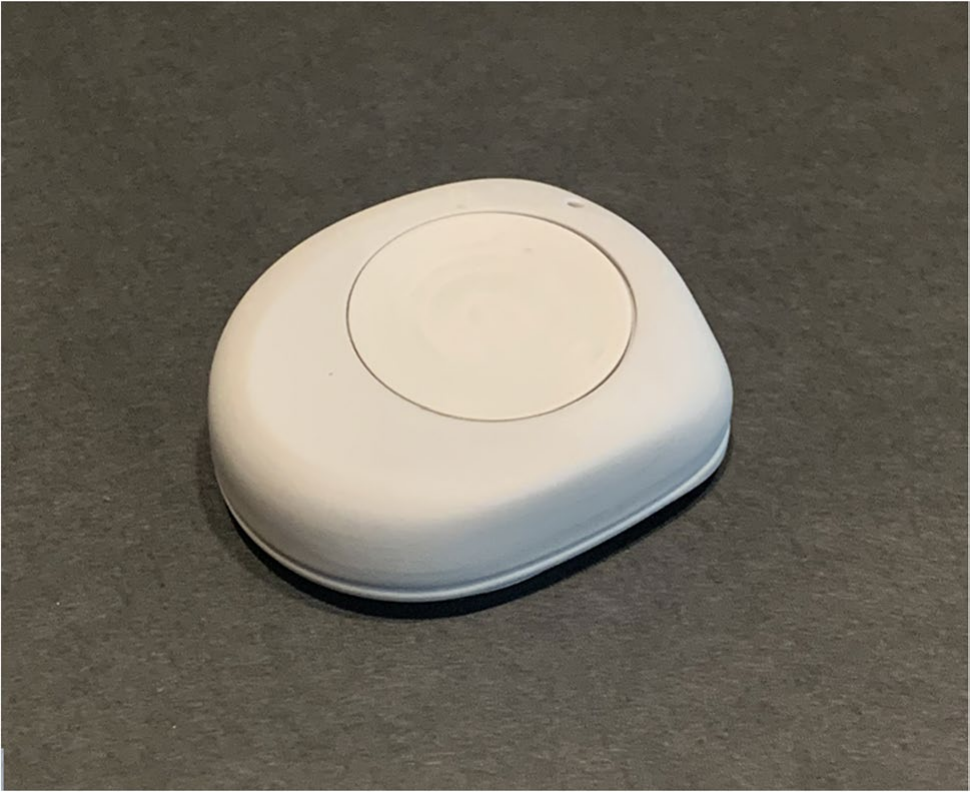
Photography of the sensor with dimensions of 33 × 39 × 13 mm and 18 g of weight

**Fig. 2:**
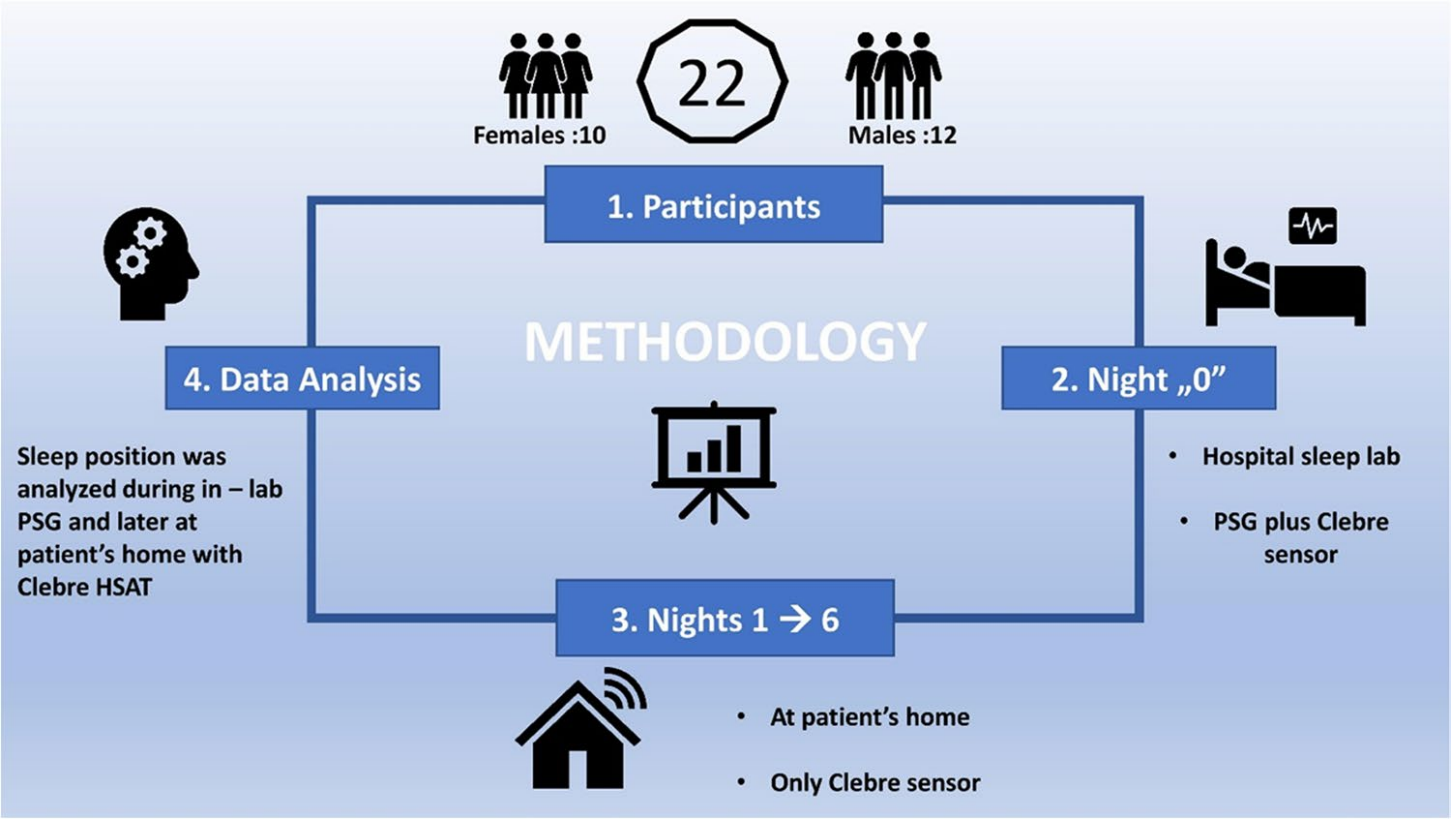
The general scheme of research methods

Subjects were given a Clebre audio and motion sensor (Fig. 1) to record from 2 to 6 nights at home in their regular bed/sleep environment. All patients were given instruction as to how to position and use the sensor. The sensor was placed by the patient in the suprasternal notch on the neck and attached using a medical double-sided patch. The dimensions of the sensor are 33 × 39 × 13 mm, and it weighs 18 g. The battery allows for at least 14 h of operation. The memory capacity is defined internally by a 2 GB FLASH chip and can be extended by an external microSD card, allowing data to be stored from several nights. A 3-axis motion accelerometry-based signals (with 52 Hz sampling frequency) estimates sleep body position characteristics, using the algorithms presented in a previous study with a 97% accuracy in supine versus non-supine body position detection compared to PSG [11]. Accuracy of body position determination in this study was calculated as 100% minus error established as a mean value of absolute differences in percentages of supine sleeping position computed by gold standard PSG and Clebre, respectively (Table 1). Instructions were given on correct use of the device by trained technicians. The Clebre sensor was given for 10–14 days during which patients were asked to record multiple at-home nights. Patients underwent PSG and Clebre in the sleep lab and Clebre for additional 2–6 nights at home (Fig. 2).

**Table 1:** Accuracy between polysomnography (PSG) and Clebre (home sleep apnea test) in determining supine sleep position in a group of 11 patients where both studies were used simultaneously on the lab night

Number of patient	1	2	3	4	5	6	7	8	9	10	11
% of supine position measured in PSG	87.2	3.5	80.8	62.6	33.4	32.6	58.9	0.3	86.7	87.3	100
% of supine position measured by Clebre	87.2	3.0	82.1	65.6	24.6	33.0	58.5	0.6	86.6	83.0	99.2
Absolute difference [%]	0	− 14	2	5	− 26	1	− 1	83	0	− 5	− 1
Accuracy* [%]	99.99	99.52	98.66	97.04	91.21	99.60	99.60	99.75	99.90	95.70	99.20
Mean accuracy [%]	98.2										

*Measured as 100% minus error established as a mean value of absolute differences in percentages of supine sleeping position

### Data analysis and statistics

For each night (in the laboratory with PSG and at home with Clebre), the percentage of supine/non-supine body position was recorded. Boxplots were used for comparison and analyzed using a paired T test (chosen after performing the Shapiro–Wilk statistical test for normality). Also, analysis of variance was conducted to assess the impact of the following factors: age (divided into 4 groups: ≤ 40, 40–50, 50–65, > 65), BMI (divided into normal: < 25, overweight: 25–30, obese: > 30), ESS (divided into lower normal: 0–5, higher normal: 6–10, mild: 11–12, moderate to severe: 13–24), and AHI (mild 5 ≤ AHI < 15, moderate 15 ≤ AHI < 30, and severe AHI ≥ 30). Signal-related calculations were performed using MATLAB 2019b (Mathworks, Natick, MA). Statistical analysis was carried out in the R Environment [14]. Visualizations were made using Python 3.7.7 and Plotly (Montreal, Canada). Significance was set at *p* < 0.05.

## Results

**Fig. 3:**
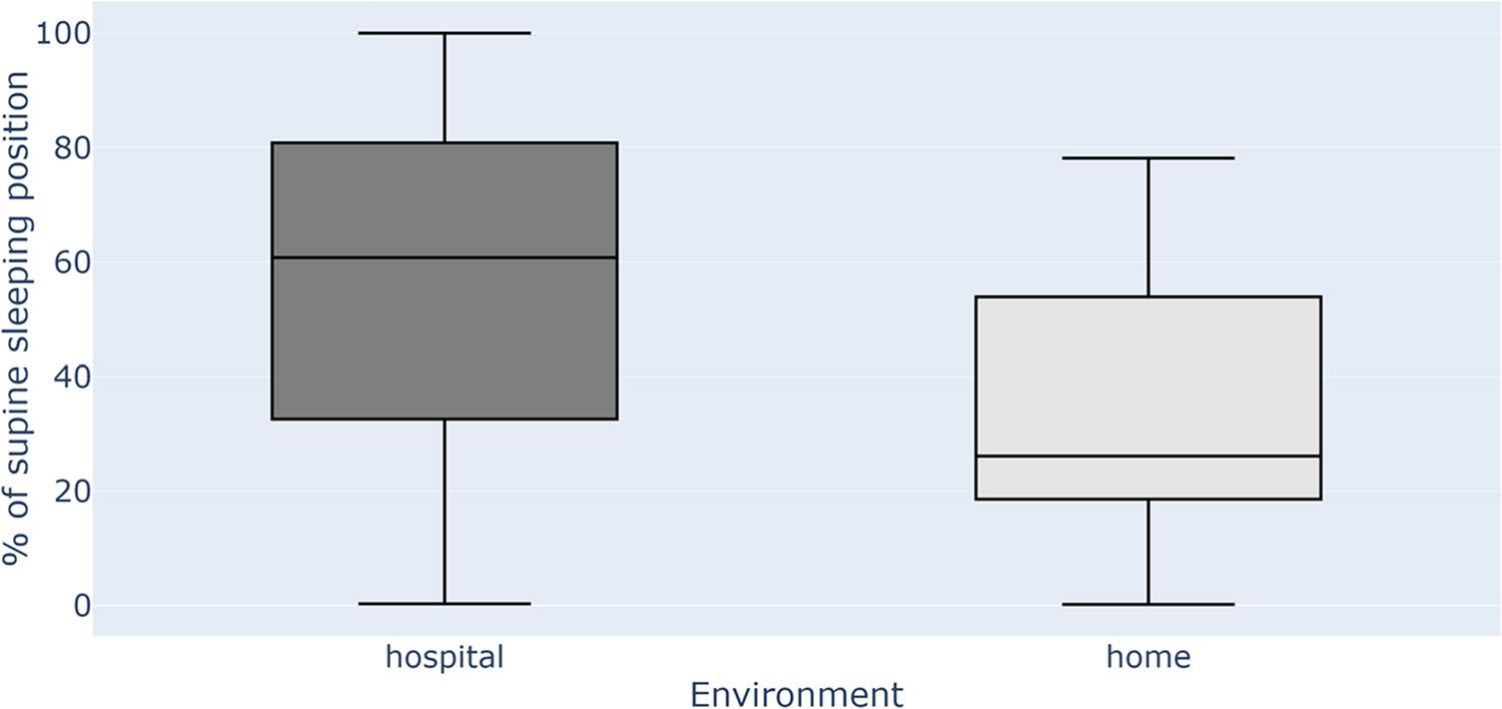
The boxplots showing median amount of supine sleep in a hospital sleep lab (night “0”) and at home (median from nights 1–6)

The study included 10 females (45%) and 12 males (55%). The median age for females and males was 60 and 45.5 years, respectively. The median AHI was 24.4 (mean 30.6), median min O_2_ saturation was 83% (mean 81.2%), and median sleep efficiency was 88.9% (mean 84.6%). The results from 11 patients confirmed the efficacy in using Clebre for sleep position determination (Table 1). The accuracy calculated in a way described in “Methods” section was 98.2%. The median percentage of supine sleep for hospital in-lab night was 61% and for home sleep was 26% (*p* < 0.001) (Fig. 3). The mean number of nights for HSAT was 3.7 (range 2–6).

**Fig. 4:**
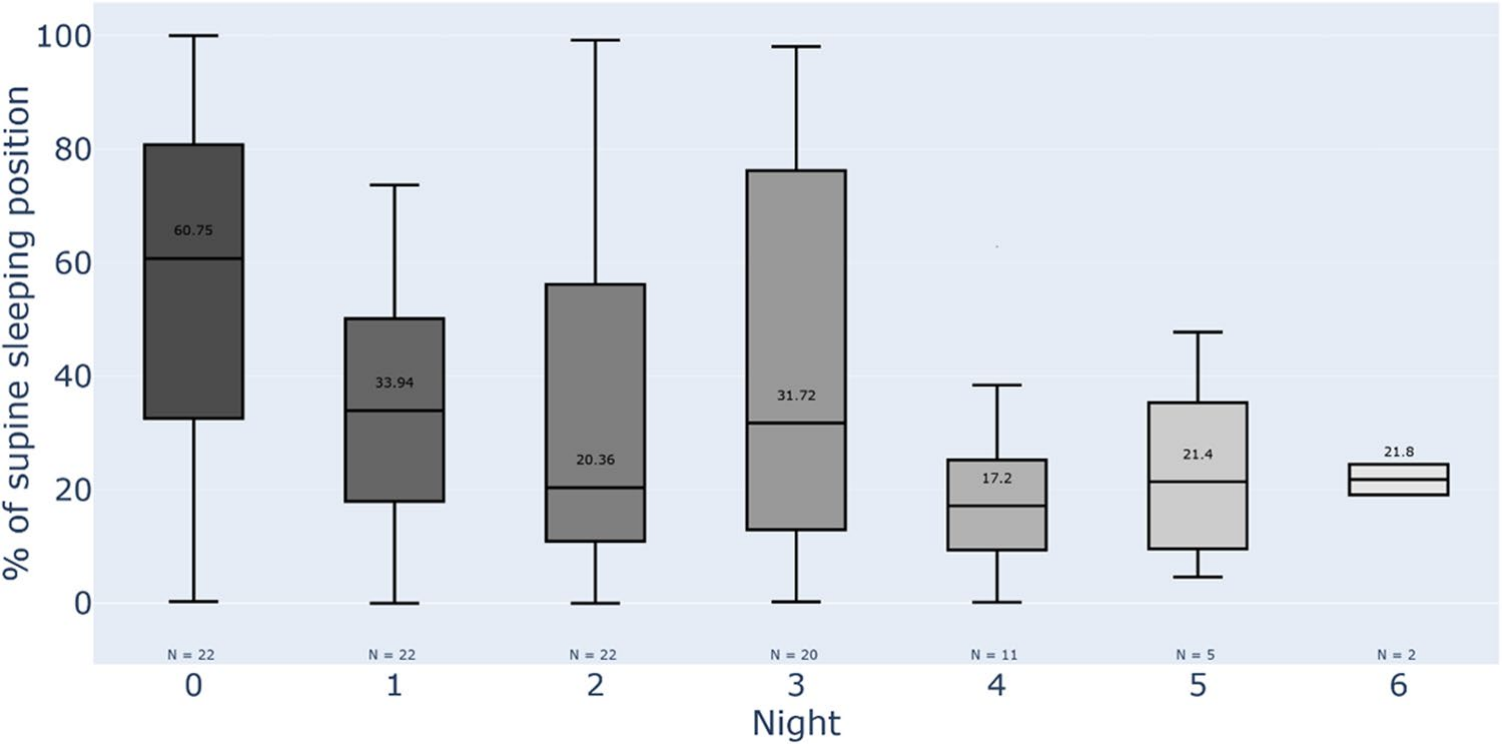
The boxplots showing median amount of supine sleep in a hospital sleep lab (night “0”) and at home from each consecutive night (1 to 6)

The median supine sleep for at-home sleep was 34% (night 1), 20% (night 2), 32% (night 3), 17% (night 4), 21% (night 5), and 22% (night 6) (Fig. 4). The analyses of variance showed no statistically significant association between AHI, BMI, ESS, sex or age groups, and percentage of supine sleep for PSG and home sleep (*p* > 0.1 for all factors).

**Fig. 5:**
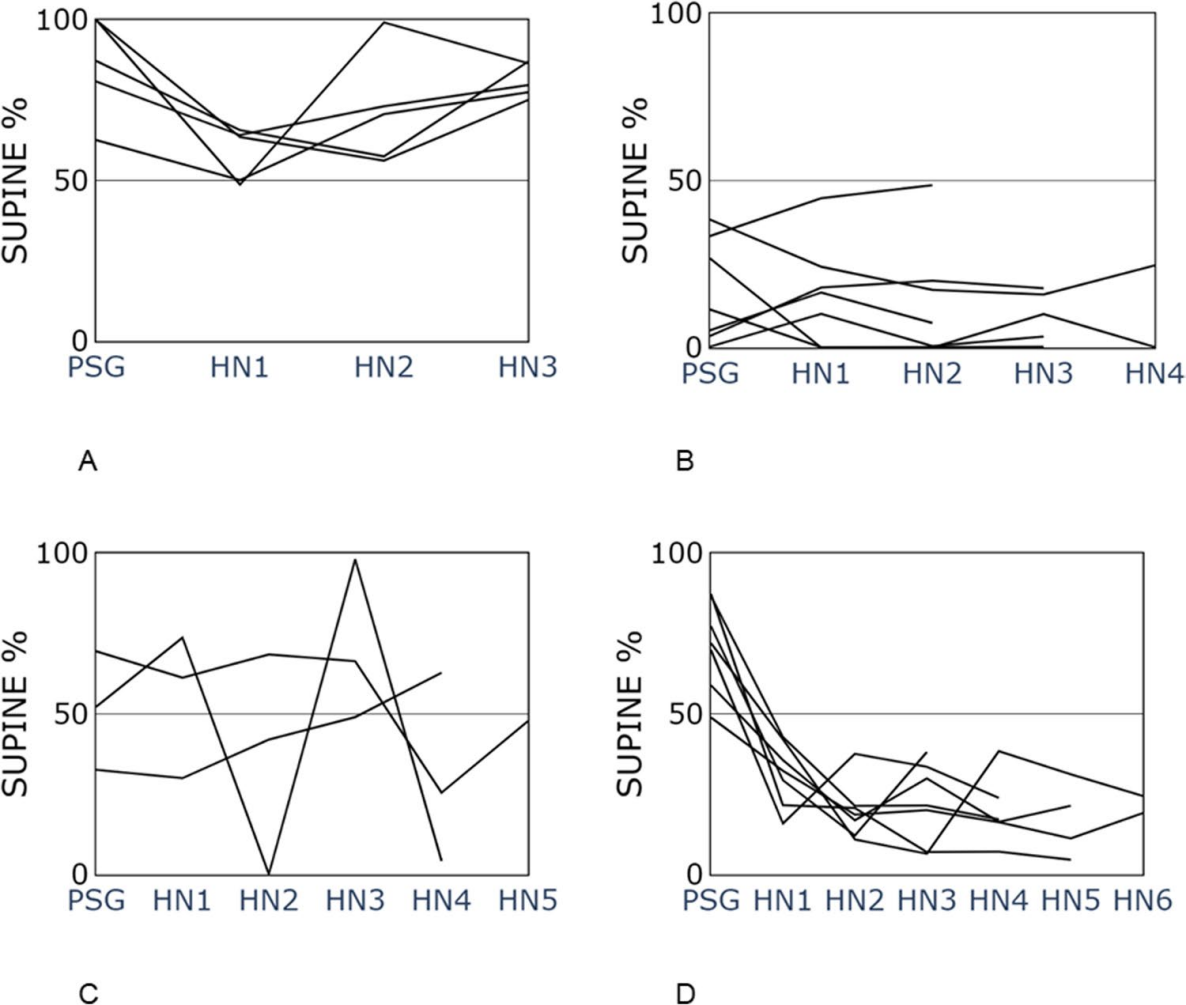
Individual supine sleep values of each of 22 patients divided into 4 groups according to their sleep pattern. “PSG” refers to in-lab hospital night “0” and HN1-HN6 to “home night” 1 to 6. **A** Patients who mainly slept supine during all nights (*n* = 5). **B** Patients who mainly slept non-supine during all nights (*n* = 7). **C** Patients who slept in different positions during each night (*n* = 3). **D** Patients who slept supine during hospital in-lab night and non-supine during at home sleep (*n* = 7)

Patients were divided into four “phenotype” groups, according to their sleep position patterns during PSG and home sleep. Group 1 slept more than 50% of time supine during all nights (*n* = 5); group 2 slept more than 50% non-supine during all nights (*n* = 7); group 3 slept in different positions (both more than 50% supine and non-supine) during nights (*n* = 3), and group 4 slept supine during PSG night and non-supine during HSAT (*n* = 7) (Fig. 5 A, B, C, and D). Demographics and PSG parameters of the study population, as well as each group, are presented in Table 2.

**Table 2:** Baseline demographic and polysomnography (PSG) data of all patients and subgroups regarding sleep position during PSG and home sleep apnea test (HSAT)

	All patients	Group 1	Group 2	Group 3	Group 4
N/sex	12 M 10F	3 M 2F	5 M 2F	0 M 3F	4 M 3F
Age (y)	54 ± 16.5	54.6 ± 17.3	47 ± 18.4	51.3 ± 15.1	61.6 ± 14.7
BMI	28.5 ± 5.3	27.2 ± 7.4	30 ± 3.8	29 ± 6.6	27.7 ± 5.2
AHI	30.6 ± 27.4	33 ± 37.3	20.2 ± 23.6	26.7 ± 17.4	41 ± 28
ESS	8.6 ± 4.7	11.4 ± 5.5	10.4 ± 3.9	6.3 ± 2.5	5.7 ± 4.2
Sleep efficiency	84.7 ± 14.3	76 ± 26.18	85.8 ± 11.7	82.4 ± 4.1	90.57 ± 3.7

*BMI* body mass index, *AHI* apnea–hypopnea index, *ESS* Epworth Sleepiness Scale

Group 1—patients sleeping more than 50% of time supine during all nights

Group 2—patients sleeping more than 50% non-supine during all nights

Group 3—patients sleeping in different positions (both more than 50% supine and non-supine) during nights

Group 4—patients sleeping supine during PSG night and non-supine during HSAT

## Discussion

Sleep position and its effects on OSA severity have been researched for years with conflicting data and results. In 1930 Johnson and colleagues published a paper entitled “In what positions do healthy people sleep?” [15]. The authors took pictures of a person asleep with a “telechron clock, which hangs beside the bed” and reported “a repertoire of more than a dozen gross postures” of sleep. A 1983 study describes 20 to 40 different sleep positions in a single night [16]. Currently four sleep positions are described that refer to trunk orientation. This decreases the number of body shifts, which is around 11 per night [17]. In the majority of patients, the severity of OSA increases while sleeping supine, and in up to 60%, AHI is 50% or greater in supine than non-supine sleep [6]. That makes it clinically important, to identify supine and non-supine sleep positions to interpret the sleep study correctly and to make sure that the proportion of supine sleep during a single night sleep study reflects natural home sleep.

In this pilot study, we describe four patterns of supine/non-supine sleep that may become important in the phenotyping process in personalizing sleep medicine. This could help in classifying patients into different prognostic and therapeutic categories [18]. Interestingly, a study by Mokros et al. showed that normal BMI has a very high negative predictive value for moderate to severe OSA in the lateral position, virtually excluding it in this position [19]. Therefore, in non-obese patients, increased supine sleep (affected by PSG equipment during a sleep study) may significantly overestimate the severity of OSA. This would be similar in the Asian population, where the prevalence of pOSA has been shown to be higher [20, 21]. Also, after upper airway surgery for OSA patients become more positional dependent. Therefore an increased supine sleep during a post-op PSG is likely to significantly affect the study results [22, 23].

In our study, group 1 and 2 have a stable proportion of supine sleep regardless of measurements in-lab or at home. In these groups, the result of PSG is reliable, at least in terms of pOSA. Group 3 shows a night-to-night variability, and group 4 shows a significant difference between PSG and home sleep. In groups 3 and 4, a single-night study is not reliable and may significantly overdiagnose OSA severity. As the amount of supine sleep is one of the important factors of night-to-night variability leading to OSA severity, group 3 patients with positional OSA would also be the individuals with the highest AHI variability during consecutive PSGs and nights [24]. Interestingly, in group 4, 6 of 7 patients had an AHI ≥ 15, and among them, 3 (50%) had positional OSA. In these three patients, the result of one night PSG could significantly overestimate the severity of OSA. This pilot study makes a strong argument against single-night sleep studies. pOSA patients from group 4 might also be mistakenly prescribed positional therapy (PT) based on one-night sleep study [9]. This type of treatment requires not only the patient to be positional-dependent, but also to sleep long enough in supine position during regular home sleep. If an individual sleeps a high proportion of the PSG night in the supine position and does not sleep supine at home, PT might be the wrong and ineffective treatment.

Another important result of this study is the difference in mean amount of supine sleep during PSG and home sleep. In a previous study, we reported retrospectively a mean supine sleep of 44% on 445 consecutive patients undergoing PSG [25]. The current study reports that mean supine sleep during PSG is 55% (median 61%) and during home sleep using Clebre HSAT is 35% (median 26%). Sorscher et al. studied how well patients can estimate supine versus non-supine sleep [26]. The mean estimated supine sleep was 20% as reported by the patient, compared to 41% during a HSAT. They concluded that patients underestimate supine sleep by up to 50%. Gordon et al. used videotaping alone on 2 nights and also reported 22% of supine sleep [17]. It is possible that patients do not underestimate supine sleep at home, as suggested by Sorscher et al., but that they simply sleep more supine during PSG due to the unfamiliar environment and use of multiple monitors.

Vonk et al. reported supine sleep in participants with positional OSA, of 43% and 29% during PSG and HSAT, respectively, using the sleep positional trainer (SPT) [27]. Wimaleswaren et al. (conference abstract) reported on 19 patients with supine sleep of 35% and 25% during PSG and HSAT, respectively, using a body position sensor (night shift) [28]. However, the accuracy of the night shift sensor has been questioned in another study reporting that in 2 out of 20 patients, night shift under-reported supine sleep, one in a situation of trunk supine and neck upright position and the other with trunk supine and head lateral position [29]. The Clebre sensor used in this study is mounted on the suprasternal notch and may be a more accurate way of measuring supine sleep at home. In a previous study, we showed a 97% accuracy of Clebre sensor in detecting supine vs non-supine sleep position compared to NOX A1 PSG [11].

There are several limitations in this study that need to be recognized and addressed in the future research. First, the sample size in this pilot study is small which may account for the lack of correlation between sleep position and AHI, BMI, ESS, sex, or age. Another major limitation is the use of new technology that requires more study but shows great promise especially for community-based research. Additionally, using PSG and Clebre in the lab may have allowed the patients to get used to sleeping with monitors and to affect future results. The sequence of monitoring should be changed for some patients in future studies. Finally, different factors such as room temperature or humidity, as well as multiple comorbidities (cardiovascular, neuromuscular, etc.), can potentially influence the sleep positions chosen by a patient and require further study.

The strength of the study is the use of a sensor in the lab and at home which enabled detection of sleep positions that were compared to PSG [11] in consecutive patients. This HSAT leads to a minimal disturbance to sleep physiology and to the ability to study the same participants in the lab and for several nights at home.

## Conclusions

We identified four distinct patterns of supine/non-supine sleep in this pilot study on 22 patients. Directing treatment by phenotype is an important move forward in the era of “personalized sleep medicine.” As there may be certain groups of patients who tend to sleep more supine during PSG than during home sleep, a one-night sleep study might be insufficient and may overestimate OSA severity in these individuals.

## Data Availability

Not applicable.
